# Crystal structure of 1-(1-chloro­eth­yl)-6,7-dimeth­oxy-1,2,3,4-tetra­hydro­isoquinolinium chloride

**DOI:** 10.1107/S2056989024011277

**Published:** 2024-11-28

**Authors:** Ziroat E. Urunbaeva, Kambarali K. Turgunov, Abdusalom Sh. Saidov, Valentina I. Vinogradova

**Affiliations:** a Samarkand State University named after Sh. Rashidov University blv 15 Samarkand 140104 Uzbekistan; b S. Yunusov Institute of the Chemistry of Plant Substances Academy of Sciences of Uzbekistan, Mirzo Ulugbek Str., 77, Tashkent 100170, Uzbekistan; c Turin Polytechnic University in Tashkent 100095 17 Little Ring Road Tashkent Uzbekistan; Universidad de la Repüblica, Uruguay

**Keywords:** crystal structure, enanti­omer, homoveratryl­amine, lactic acid, tetra­hydro­iso­quinoline

## Abstract

1-(1-Chloro­eth­yl)-6,7-dimeth­oxy-1,2,3,4-tetra­hydro­iso­quinoline was synthesized through the reaction of homoveratryl­amine with racemic lactic acid. The formation of two enanti­omers, *RR* and *SS*, was detected by performing X-ray diffraction analysis on their chloride salts. The asymmetric unit of the crystal consists of a C_13_H_19_ClNO_2_^+^ mol­ecular cation and a Cl^−^ anion. Two protonated enanti­omers of the title compound, with *RR* and *SS* configurations of the chiral atoms, are connected into hydrogen-bonded dimers bridged by Cl^−^ anions. Weak C—H⋯Cl inter­actions lead to the formation of a chain running along the *a-*axis direction of the unit cell, which corresponds to the longest dimension (the preferential growth direction) of the needle-shaped monocrystal. The crystal studied was refined as a two-component twin.

## Abstract 2

The title compound crystallizes in the monoclinic *P*2_1_/*c* (No. 14) space group. The asymmetric unit of the crystal contains one independent mol­ecule with an *1R*, *11R* configuration of chiral carbon atoms.

## 1. Chemical context

Iso­quinoline alkaloids, widely distributed in the plant and animal kingdoms, have received much attention because of their important biological activities (Lundstorom, 1983 ▸). For example, 1.2.3.4-tetra­hydro­iso­quinoline and 2-methyl-1.2.3.4-tetra­hydro­iso­quinoline, present in mammalian brains, are known to induce Parkinson’s disease (Ohta *et al.*, 1987 ▸; Niwa *et al.* 1987 ▸). Effective synthetic methods for preparing of 1.2.3.4-tetra­hydro­iso­quinoline derivatives have been found (Shinohara *et al.* 1997 ▸). 1-Substituted-1,2,3,4-tetra­hydro­iso­quinolines are especially intriguing among the synthetic derivatives of the iso­quinoline alkaloid. They feature biologically active compounds, for example, an anti­epileptic agent (Gitto *et al.*, 2003 ▸) and a derivative with inhibitory activity against bladder contraction (Naito *et al.*, 2005 ▸). A lot of work has been done on the synthesis and structural studies of 1-substituted-1,2,3,4-tetra­hydro­iso­quinolines in search of active compounds (Olszak *et al.*, 1996 ▸; Pashev *et al., 2020 ▸;* Turgunov *et al.* 2016 ▸).

**Figure 1:**
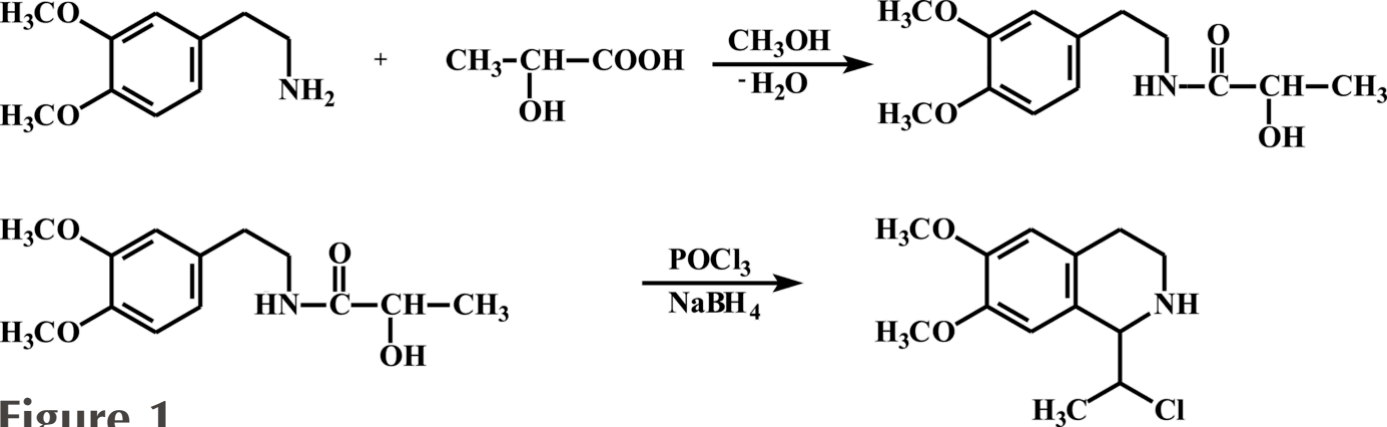
Synthesis scheme of the title compound.

In this context, we treated homoveratryl­amine with lactic acid and obtained the corresponding amide inter­mediate. Cyclization of the amide with POCl_3_ and NaBH_4_ afforded the title compound (Fig. 1 ▸). Racemic lactic acid was used in the synthesis, so four stereoisomers (*R,R; R,S; S,S; S,R*) of 1-(1-chloro­eth­yl)-6,7-dimeth­oxy-1,2,3,4-tetra­hydro­iso­quinoline were expected. Currently, we have detected the formation of two enanti­omers, *RR* and *SS*, packed in a single crystal by X-ray diffraction (XRD) analysis. A detailed analysis of the reaction products is ongoing and will be published in our future work. To obtain good crystals suitable for XRD analysis, hydro­chlorides of the iso­quinolines were used.
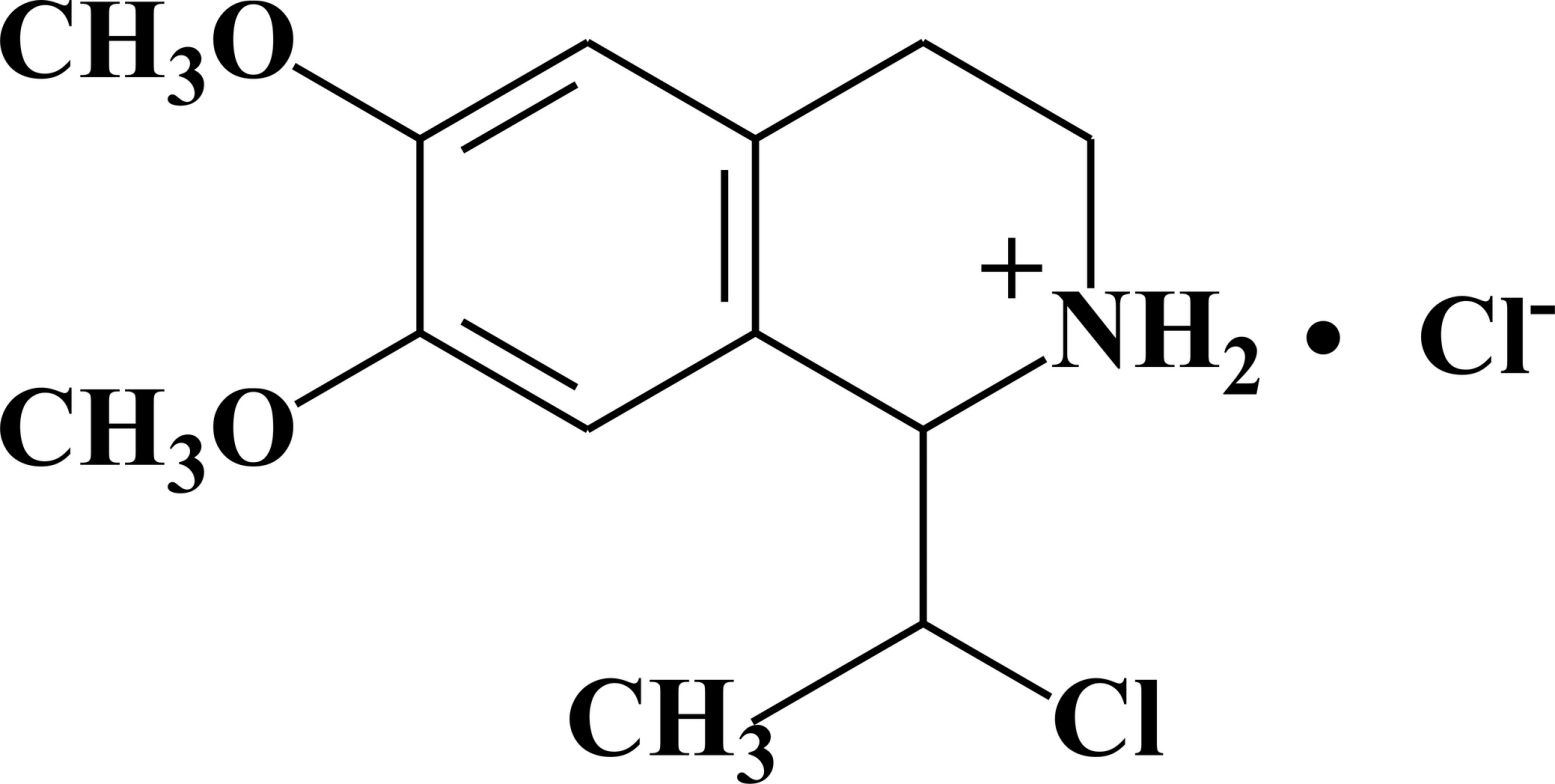


## 2. Structural commentary

**Figure 2:**
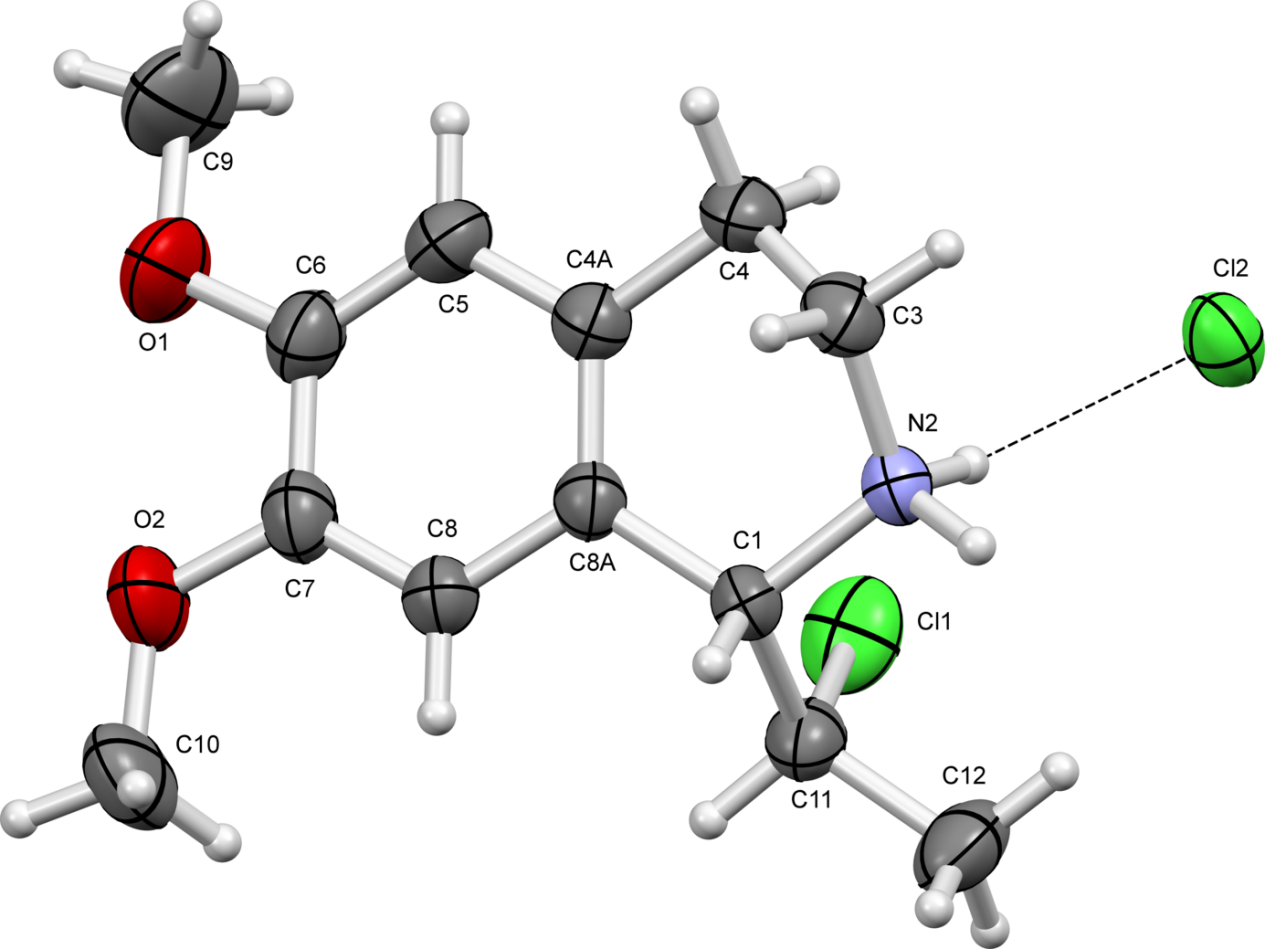
Displacement ellipsoid plot of the title compound with atom labels. Ellipsoids are drawn at the 50% probability level. The hydrogen bond formed between the mol­ecular cation and the chlorine anion is showed as a dashed line.

The title compound crystallizes in the monoclinic *P*2_1_/*c* (No. 14) space group. The asymmetric unit of the crystal contains one independent mol­ecule with an *1S*, *11S* configuration of chiral carbon atoms, so the crystal consists of *RR* and *SS* enanti­omers. The C4*A*/C4–C8/C8*A* aromatic ring is twisted slightly with a slightly high value for the r.m.s. deviation (0.0245 Å) of the fitted atoms from the mean plane of the ring. The meth­oxy groups at C6 and C7 atoms are slightly rotated around the C6—O1 and C7—O2 bonds (Fig. 2 ▸), the dihedral angles between the plane of the aromatic ring and the planes defined by atoms C6/O1/C9 and C7/O2/C10 being 13.0 (3) and 6.5 (3)°, respectively. The C4*A*—C4 and C8*A–*-C1 bonds are slightly out of the plane, the deviations of C1 and C4 from the mean plane of aromatic ring being 0.206 (2) and −0.147 (2) Å, respectively. The heterocyclic ring of tetra­hydro­iso­quinoline adopts a half chair conformation.

## 3. Supra­molecular features

**Figure 3:**
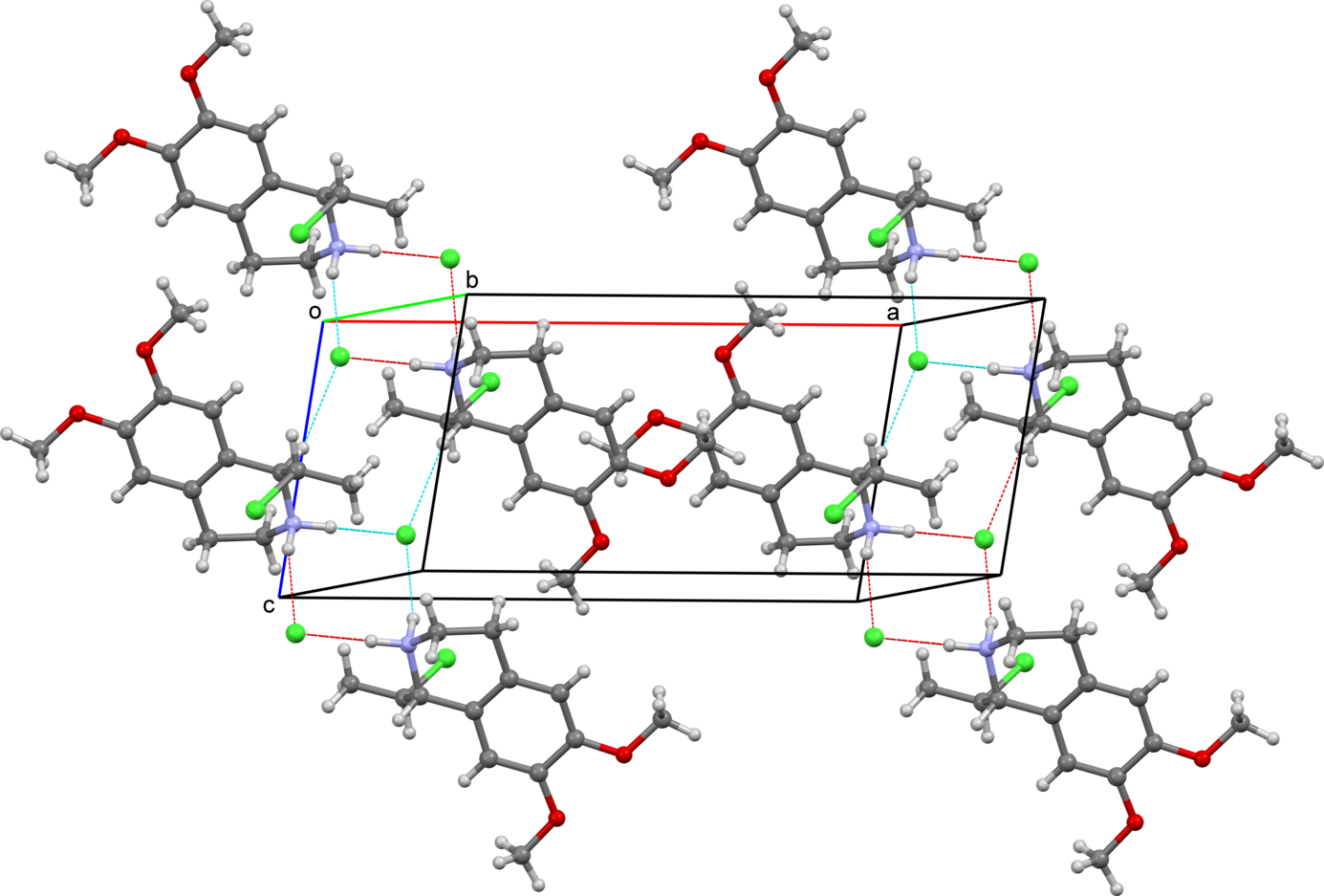
Hydrogen bonding in the crystal of the title compound.

The presence of both enanti­omers of the title compound in the crystal allows the mol­ecules to link into inversion dimers through N2—H2*A*⋯Cl2 and N2—H2*B*⋯Cl2^i^ [symmetry code: (i) 2 − *x*, 1 − *y*, 2 − *z*] inter­molecular inter­actions, forming rings with the graph-set motif 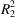
(8) (Fig. 3 ▸, Table 1 ▸) where the Cl2 anions act as double hydrogen-bond acceptors. In addition, pairs of C1—H1*A*⋯Cl2 weak inter­actions lead to chain formation along the *a-*axis direction, which is the longest cell dimension (preferential growth direction) of the monocrystal. A C12—H12*A*⋯Cl2 weak inter­action leads to the formation of hydrogen-bonded layers parallel to the *bc* plane.

## 4. Database survey

A search in the Cambridge Structural Database (CSD, version 5.43, update of November 2022; Groom *et al.*, 2016 ▸) revealed 123 structures of 1-substituted and 2-substituted 6,7-dimeth­oxy-1,2,3,4-tetra­hydro­iso­quinolines. Among these, 15 structures correspond to 1-substituted 6,7-dimeth­oxy-1,2,3,4-tetra­hydro­iso­quinolines. Enanti­opure crystal structures were determined for (*R*)-1-hy­droxy­methyl-6,7-dimeth­oxy-1,2,3,4-tetra­hydro­iso­quinoline (refcode: BIMCEG) and (*S*)-1-hy­droxy­methyl-6,7-dimeth­oxy-1,2,3,4-tetra­hydro­iso­quinoline chloride (refcode: BIMCIK), alkaloids isolated from seeds of *Calycotome Villosa* (Antri *et al.*, 2004 ▸). A search in the Cambridge Structural Database for the cationic form of 6,7-dimeth­oxy-1,2,3,4-tetra­hydro­iso­quinoline resulted in 13 hits. Ten of these, where the mol­ecule contains a chiral atom, are enanti­opure crystals containing only proper symmetry elements. Therefore, in these crystal structures, the inter­linking of mol­ecules by hydrogen bonds differs from our case.

## 5. Synthesis and crystallization

*N-(3,4-Di­meth­oxy­phenyl­eth­yl)-2-hy­droxy­propanamide*. A mixture of 1.81 g (0.01 mol) of homoveratrilamine and 0.9 g (0.01 mol) of lactic acid was dissolved in 5 ml of methanol. Self-heating occurred. Then the mixture was heated in an oil bath for 2 h at a temperature of 451–453 K. The progress of the reaction was monitored by TLC. The reaction mixture was dissolved in 100 mL of chloro­form. The chloro­form layer was first washed three times with 3% hydro­chloric acid. The chloro­form solution was then washed with water until neutral, followed by washing with 2% sodium hydroxide solution and water until neutral. The resulting chloro­form solution was dried over Na_2_SO_4_ and then evaporated. The residue was crystallized from a mixture (acetone-hexa­ne). White crystals with m.p. 343–344 K. Yield 70% (1.77 g). *R*_f_ = 0.40 chloro­form-methanol (8:2).

^1^H NMR: (400 MHz, CDCl_3_, δ, ppm., *J*/Hz): 1.34 (3H, *d*, *J* = 6.7, H-3′), 2.73 (2H, *t*, *J* = 7.1, H-α), 3.46 (2H, *q*, *J* = 6.7, H-β), 3.81 (3H, *s*, OCH_3_), 3.82 (3H, *s*, OCH_3_), 4.10 (1H, *wide s*, OH), 4.17 (1H, *q*, *J* = 6.8 H −2′), 6.69 (2H, *top – top*, H-2,6), 6.77 (1H, *d*, *J* = 8.6, H-5), 6.90 (1H, *wide s*, NH).

^13^C NMR: 21.26 C-3, 35.19 C-α; 40.60 C-β, 55.96 C-OCH_3_, 55.96 C-OCH_3_, 68.10 C-2I, 111.44 C-2, 111.97 C-5, 131 C-1, 120.73 C-6, 147.73 C-3, 149.01 C-4, 175.59 C-1-CO.

MS *m*/*z* (*M*^+^) 253, 224, 165, 123.9 (124), 59.8 (60).

*1-(1-Chloro­eth­yl)-6,7-dimeth­oxy-1,2,3,4-tetra­hydro­iso­quin­oline.* 1.550 mg (0.0061 mol) of *N*-(3,4-di­meth­oxy­phenyl­eth­yl)-2-hy­droxy­propanamide were dissolved in 30 ml of absolute benzene, then 0.9404 mg (0.0061 mol) or 0.6–1 ml of POCl_3_ were added. The reaction mixture was refluxed with a calcium chloride tube for 2 h. The progress of the reaction was monitored by TLC. After the reaction was complete after 2.5 h, benzene and residual POCl_3_ were removed and the residue was dried. The residue was then dissolved in 50 mL of methanol. 0.6 g of NaBH_4_ was added in small portions at 273–323 K for 3 h with constant stirring. This mixture was left overnight. The solvent was then removed and the residue was dissolved in distilled water. The aqueous layer was extracted several times with chloro­form. The chloro­form layer was combined and washed with water. After that, the chloro­form layer was dried with Na_2_SO_4_. The residue was dissolved in methanol and precipitated as the hydro­chloride using concentrated HCl solution. The precipitate was filtered, washed in acetone and dried. Yield 0.843 g (59%) (0.843 g), m.p. 476–477 K, *R*_f_ = 0.57 (chloro­form–methanol 8:1.5).

^1^H NMR (400 MHz, CDCl_3_, δ, ppm, *J* / Hz): 1.87 (3H, *d*, *J* = 7, H-3′), 2.91 (2H, *m*, H-3a), 3.23 (2H, *m*, H-4), 3.84 (1H, *m*, H-3e), 3.85 (3H, *s*, OCH_3_), 3.86 (3H, *s*, OCH_3_), 4.59 (1H, *q*, *J* = 3.5, H-2′), 4.74 (1H, *d*, *J* = 3.3, H-1), 6.61 (1H, *s*, H-8), 6.69 (1H, *s*, H-5).

### 5.1. Refinement

Crystal data, data collection and structure refinement details are summarized in Table 2 ▸. The crystal under investigation exhibited twinning, which was identified during the initial analysis of the diffraction data. The twin law was determined based on the symmetry of the crystal and the diffraction analysis. In this case, a twofold rotation axis **(**along the *c* axis) related the two twin domains, with each domain contributing to the overall diffraction pattern. The twin fraction was estimated to be approximately 0.60 for component 1 and 0.40 for component 2, based on the refinement of the intensity data. Reflections in the HKLF 5 format were used for structure determination and refinement. The H atoms bonded to C atoms were placed geometrically (with C—H distances of 0.98 Å for CH, 0.97 Å for CH_2_, 0.96 Å for CH_3_ and 0.93 Å for C_ar_) and included in the refinement in a riding-motion approximation with *U*_iso_(H) = 1.2*U*_eq_(C) [*U*_iso_ = 1.5*U*_eq_(C) for methyl H atoms]. The hydrogen atoms on the N1 were located in difference-Fourier maps and refined freely.

## Supplementary Material

Crystal structure: contains datablock(s) I, GLOBAL. DOI: 10.1107/S2056989024011277/ny2008sup1.cif

Structure factors: contains datablock(s) I. DOI: 10.1107/S2056989024011277/ny2008Isup2.hkl

Supporting information file. DOI: 10.1107/S2056989024011277/ny2008Isup3.cml

CCDC reference: 2403944

Additional supporting information:  crystallographic information; 3D view; checkCIF report

## Figures and Tables

**Table 1:** Hydrogen-bond geometry (Å, °)

*D*—H⋯*A*	*D*—H	H⋯*A*	*D*⋯*A*	*D*—H⋯*A*
C1—H1*A*⋯Cl2^i^	0.98	2.62	3.450 (2)	143
N2—H2*A*⋯Cl2^ii^	0.95 (2)	2.14 (2)	3.0895 (19)	177 (2)
N2—H2*B*⋯Cl1	0.92 (2)	2.75 (3)	3.1828 (19)	110 (2)
N2—H2*B*⋯Cl2	0.92 (2)	2.27 (2)	3.0751 (19)	146 (2)
C11—H11*A*⋯Cl1^iii^	0.98	2.93	3.767 (2)	144
C12—H12*A*⋯Cl2^iii^	0.96	2.75	3.710 (3)	174

Symmetry codes: (i) 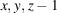; (ii) 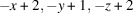; (iii) 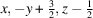.

**Table 2:** Experimental details

Crystal data	
Chemical formula	C_13_H_19_ClNO_2_^+^·Cl^−^
*M*_r_	292.19
Crystal system, space group	Monoclinic, *P*2_1_/*c*
Temperature (K)	293
*a*, *b*, *c* (Å)	16.1298 (3), 12.3736 (3), 7.46745 (16)
β (°)	100.190 (2)
*V* (Å^3^)	1466.87 (6)
*Z*	4
Radiation type	Cu *K*α
μ (mm^−1^)	3.94
Crystal size (mm)	0.25 × 0.10 × 0.05

Data collection	
Diffractometer	XtaLAB Synergy, Single source at home/near, HyPix3000
Absorption correction	Multi-scan (*CrysAlis PRO*; Rigaku OD, 2018 ▸)
*T*_min_, *T*_max_	0.784, 1.000
No. of measured, independent and observed [*I* > 2σ(*I*)] reflections	12790, 4641, 4142
*R*_int_	0.061
(sin θ/λ)_max_ (Å^−1^)	0.602

Refinement	
*R*[*F*^2^ > 2σ(*F*^2^)], *wR*(*F*^2^), *S*	0.050, 0.155, 1.09
No. of reflections	4641
No. of parameters	170
No. of restraints	2
H-atom treatment	H atoms treated by a mixture of independent and constrained refinement
Δρ_max_, Δρ_min_ (e Å^−3^)	0.32, −0.26

Computer programs: *CrysAlis PRO* (Rigaku OD, 2018 ▸), *SHELXT2018/2* (Sheldrick, 2015*a* ▸), *SHELXL2018/3* (Sheldrick, 2015*b* ▸), *Mercury* (Macrae *et al.*, 2020 ▸), *SHELXL2014/7* (Sheldrick, 2015*b* ▸).

## supplementary crystallographic information

### 1-(1-Chloroethyl)-6,7-dimethoxy-1,2,3,4-tetrahydroisoquinolinium chloride Crystal data

**Table:**

C_13_H_19_ClNO_2_^+^·Cl^−^	*D*_x_ = 1.323 Mg m^−^^3^
*M**_r_* = 292.19	Melting point: 476(2) K
Monoclinic, *P*2_1_/*c*	Cu *K*α radiation, λ = 1.54184 Å
*a* = 16.1298 (3) Å	Cell parameters from 5593 reflections
*b* = 12.3736 (3) Å	θ = 2.8–68.0°
*c* = 7.46745 (16) Å	µ = 3.94 mm^−^^1^
β = 100.190 (2)°	*T* = 293 K
*V* = 1466.87 (6) Å^3^	Prism, colourless
*Z* = 4	0.25 × 0.10 × 0.05 mm
*F*(000) = 616	

### 1-(1-Chloroethyl)-6,7-dimethoxy-1,2,3,4-tetrahydroisoquinolinium chloride Data collection

**Table:**

XtaLAB Synergy, Single source at home/near, HyPix3000 diffractometer	4641 independent reflections
Radiation source: micro-focus sealed X-ray tube, PhotonJet (Cu) X-ray Source	4142 reflections with *I* > 2σ(*I*)
Mirror monochromator	*R*_int_ = 0.061
Detector resolution: 10.0000 pixels mm^-1^	θ_max_ = 68.2°, θ_min_ = 7.0°
wσcans	*h* = −17→19
Absorption correction: multi-scan (CrysAlisPro; Rigaku OD, 2018)	*k* = −14→14
*T*_min_ = 0.784, *T*_max_ = 1.000	*l* = −8→7
12790 measured reflections	

### 1-(1-Chloroethyl)-6,7-dimethoxy-1,2,3,4-tetrahydroisoquinolinium chloride Refinement

**Table:**

Refinement on *F*^2^	Primary atom site location: iterative
Least-squares matrix: full	Secondary atom site location: difference Fourier map
*R*[*F*^2^ > 2σ(*F*^2^)] = 0.050	Hydrogen site location: mixed
*wR*(*F*^2^) = 0.155	H atoms treated by a mixture of independent and constrained refinement
*S* = 1.09	*w* = 1/[σ^2^(*F*_o_^2^) + (0.1122*P*)^2^ + 0.0537*P*] where *P* = (*F*_o_^2^ + 2*F*_c_^2^)/3
4641 reflections	(Δ/σ)_max_ < 0.001
170 parameters	Δρ_max_ = 0.32 e Å^−^^3^
2 restraints	Δρ_min_ = −0.26 e Å^−^^3^

### 1-(1-Chloroethyl)-6,7-dimethoxy-1,2,3,4-tetrahydroisoquinolinium chloride Special details

**Table:**

Geometry. All esds (except the esd in the dihedral angle between two l.s. planes) are estimated using the full covariance matrix. The cell esds are taken into account individually in the estimation of esds in distances, angles and torsion angles; correlations between esds in cell parameters are only used when they are defined by crystal symmetry. An approximate (isotropic) treatment of cell esds is used for estimating esds involving l.s. planes.
Refinement. Refined as a 2-component twin

### 1-(1-Chloroethyl)-6,7-dimethoxy-1,2,3,4-tetrahydroisoquinolinium chloride Fractional atomic coordinates and isotropic or equivalent isotropic displacement parameters (Å^2^)

**Table:**

	*x*	*y*	*z*	*U*_iso_*/*U*_eq_	
Cl1	0.77537 (4)	0.66403 (6)	0.75105 (11)	0.0673 (3)	
Cl2	0.91248 (3)	0.52447 (5)	1.18130 (7)	0.0506 (2)	
O1	0.51533 (12)	0.3579 (2)	0.3763 (3)	0.0747 (6)	
O2	0.58615 (12)	0.47214 (18)	0.1566 (3)	0.0644 (6)	
C1	0.85038 (13)	0.49829 (17)	0.5981 (3)	0.0358 (5)	
H1A	0.884823	0.477867	0.507792	0.043*	
N2	0.89415 (11)	0.45706 (15)	0.7799 (2)	0.0374 (4)	
H2A	0.9536 (13)	0.465 (2)	0.791 (4)	0.045*	
H2B	0.8830 (17)	0.498 (2)	0.876 (3)	0.045*	
C3	0.87520 (15)	0.34188 (19)	0.8137 (3)	0.0465 (5)	
H3A	0.908683	0.318072	0.927730	0.056*	
H3B	0.889133	0.296935	0.716790	0.056*	
C4	0.78257 (15)	0.3314 (2)	0.8215 (3)	0.0496 (6)	
H4A	0.770169	0.370253	0.926464	0.060*	
H4B	0.768603	0.255903	0.834578	0.060*	
C4A	0.72980 (14)	0.37668 (19)	0.6497 (3)	0.0416 (5)	
C5	0.64592 (15)	0.3445 (2)	0.5957 (3)	0.0503 (6)	
H5A	0.621781	0.297843	0.669519	0.060*	
C6	0.59837 (15)	0.3808 (2)	0.4351 (4)	0.0512 (6)	
C7	0.63650 (15)	0.4458 (2)	0.3182 (3)	0.0473 (6)	
C8	0.71844 (14)	0.47887 (19)	0.3714 (3)	0.0414 (5)	
H8A	0.743570	0.522290	0.294466	0.050*	
C8A	0.76461 (13)	0.44768 (17)	0.5409 (3)	0.0369 (5)	
C9	0.4704 (2)	0.3114 (4)	0.5026 (6)	0.1007 (14)	
H9A	0.413028	0.299237	0.445017	0.151*	
H9B	0.495855	0.243834	0.544775	0.151*	
H9C	0.471544	0.359488	0.603867	0.151*	
C10	0.6234 (2)	0.5306 (3)	0.0284 (4)	0.0711 (9)	
H10A	0.581969	0.544019	−0.078361	0.107*	
H10B	0.644673	0.598139	0.080729	0.107*	
H10C	0.668884	0.489221	−0.004100	0.107*	
C11	0.84659 (14)	0.62126 (19)	0.6022 (3)	0.0455 (5)	
H11A	0.822722	0.645643	0.478997	0.055*	
C12	0.93059 (18)	0.6771 (2)	0.6574 (4)	0.0650 (7)	
H12A	0.922258	0.753986	0.656226	0.097*	
H12B	0.955872	0.654494	0.777642	0.097*	
H12C	0.966957	0.658458	0.573374	0.097*	

### 1-(1-Chloroethyl)-6,7-dimethoxy-1,2,3,4-tetrahydroisoquinolinium chloride Atomic displacement parameters (Å^2^)

**Table:**

	*U*^11^	*U*^22^	*U*^33^	*U*^12^	*U*^13^	*U*^23^
Cl1	0.0646 (4)	0.0535 (4)	0.0826 (5)	0.0072 (3)	0.0094 (4)	−0.0177 (3)
Cl2	0.0439 (3)	0.0715 (5)	0.0365 (3)	0.0006 (2)	0.0075 (2)	−0.0008 (2)
O1	0.0424 (9)	0.0996 (16)	0.0760 (12)	−0.0225 (10)	−0.0063 (9)	0.0100 (12)
O2	0.0483 (10)	0.0862 (15)	0.0512 (11)	−0.0048 (9)	−0.0120 (9)	0.0104 (9)
C1	0.0369 (10)	0.0382 (11)	0.0306 (10)	−0.0027 (8)	0.0019 (8)	0.0023 (8)
N2	0.0359 (9)	0.0418 (10)	0.0326 (9)	−0.0012 (7)	0.0008 (7)	0.0016 (7)
C3	0.0476 (12)	0.0411 (13)	0.0471 (12)	0.0041 (9)	−0.0015 (10)	0.0066 (9)
C4	0.0517 (13)	0.0465 (14)	0.0478 (12)	−0.0070 (10)	0.0009 (10)	0.0129 (10)
C4A	0.0426 (11)	0.0403 (12)	0.0405 (11)	−0.0027 (9)	0.0040 (9)	0.0012 (9)
C5	0.0455 (12)	0.0496 (14)	0.0552 (13)	−0.0112 (10)	0.0073 (10)	0.0035 (11)
C6	0.0390 (11)	0.0559 (15)	0.0555 (13)	−0.0083 (10)	0.0000 (10)	−0.0027 (11)
C7	0.0428 (11)	0.0532 (14)	0.0418 (12)	−0.0003 (10)	−0.0036 (9)	−0.0009 (10)
C8	0.0396 (11)	0.0455 (13)	0.0377 (11)	−0.0032 (9)	0.0032 (9)	0.0009 (9)
C8A	0.0371 (10)	0.0367 (11)	0.0353 (10)	−0.0010 (8)	0.0022 (8)	−0.0024 (8)
C9	0.0469 (16)	0.137 (4)	0.114 (3)	−0.030 (2)	0.0021 (18)	0.028 (3)
C10	0.0628 (17)	0.096 (2)	0.0488 (15)	0.0066 (16)	−0.0060 (13)	0.0178 (14)
C11	0.0465 (11)	0.0380 (12)	0.0482 (12)	−0.0029 (9)	−0.0019 (9)	0.0039 (9)
C12	0.0558 (15)	0.0462 (15)	0.0872 (19)	−0.0150 (12)	−0.0029 (14)	0.0039 (13)

### 1-(1-Chloroethyl)-6,7-dimethoxy-1,2,3,4-tetrahydroisoquinolinium chloride Geometric parameters (Å, º)

**Table:**

Cl1—C11	1.813 (3)	C4A—C5	1.399 (3)
O1—C6	1.364 (3)	C5—C6	1.379 (4)
O1—C9	1.410 (4)	C5—H5A	0.9300
O2—C7	1.369 (3)	C6—C7	1.407 (4)
O2—C10	1.416 (4)	C7—C8	1.374 (3)
C1—N2	1.504 (3)	C8—C8A	1.405 (3)
C1—C8A	1.510 (3)	C8—H8A	0.9300
C1—C11	1.523 (3)	C9—H9A	0.9600
C1—H1A	0.9800	C9—H9B	0.9600
N2—C3	1.488 (3)	C9—H9C	0.9600
N2—H2A	0.95 (2)	C10—H10A	0.9600
N2—H2B	0.92 (2)	C10—H10B	0.9600
C3—C4	1.511 (3)	C10—H10C	0.9600
C3—H3A	0.9700	C11—C12	1.512 (3)
C3—H3B	0.9700	C11—H11A	0.9800
C4—C4A	1.516 (3)	C12—H12A	0.9600
C4—H4A	0.9700	C12—H12B	0.9600
C4—H4B	0.9700	C12—H12C	0.9600
C4A—C8A	1.382 (3)		
			
C6—O1—C9	117.5 (2)	C5—C6—C7	119.3 (2)
C7—O2—C10	117.4 (2)	O2—C7—C8	125.1 (2)
N2—C1—C8A	112.04 (17)	O2—C7—C6	115.3 (2)
N2—C1—C11	109.57 (17)	C8—C7—C6	119.7 (2)
C8A—C1—C11	112.46 (18)	C7—C8—C8A	120.6 (2)
N2—C1—H1A	107.5	C7—C8—H8A	119.7
C8A—C1—H1A	107.5	C8A—C8—H8A	119.7
C11—C1—H1A	107.5	C4A—C8A—C8	119.9 (2)
C3—N2—C1	113.60 (16)	C4A—C8A—C1	123.00 (18)
C3—N2—H2A	108.8 (15)	C8—C8A—C1	117.02 (19)
C1—N2—H2A	110.2 (16)	O1—C9—H9A	109.5
C3—N2—H2B	108.3 (17)	O1—C9—H9B	109.5
C1—N2—H2B	113.2 (17)	H9A—C9—H9B	109.5
H2A—N2—H2B	102 (2)	O1—C9—H9C	109.5
N2—C3—C4	108.83 (18)	H9A—C9—H9C	109.5
N2—C3—H3A	109.9	H9B—C9—H9C	109.5
C4—C3—H3A	109.9	O2—C10—H10A	109.5
N2—C3—H3B	109.9	O2—C10—H10B	109.5
C4—C3—H3B	109.9	H10A—C10—H10B	109.5
H3A—C3—H3B	108.3	O2—C10—H10C	109.5
C3—C4—C4A	110.28 (19)	H10A—C10—H10C	109.5
C3—C4—H4A	109.6	H10B—C10—H10C	109.5
C4A—C4—H4A	109.6	C12—C11—C1	115.2 (2)
C3—C4—H4B	109.6	C12—C11—Cl1	109.50 (19)
C4A—C4—H4B	109.6	C1—C11—Cl1	109.62 (16)
H4A—C4—H4B	108.1	C12—C11—H11A	107.4
C8A—C4A—C5	119.0 (2)	C1—C11—H11A	107.4
C8A—C4A—C4	120.46 (19)	Cl1—C11—H11A	107.4
C5—C4A—C4	120.6 (2)	C11—C12—H12A	109.5
C6—C5—C4A	121.2 (2)	C11—C12—H12B	109.5
C6—C5—H5A	119.4	H12A—C12—H12B	109.5
C4A—C5—H5A	119.4	C11—C12—H12C	109.5
O1—C6—C5	125.2 (2)	H12A—C12—H12C	109.5
O1—C6—C7	115.6 (2)	H12B—C12—H12C	109.5
			
C8A—C1—N2—C3	34.0 (2)	C5—C6—C7—C8	−4.8 (4)
C11—C1—N2—C3	159.49 (19)	O2—C7—C8—C8A	179.7 (2)
C1—N2—C3—C4	−64.4 (2)	C6—C7—C8—C8A	0.5 (4)
N2—C3—C4—C4A	55.1 (3)	C5—C4A—C8A—C8	−5.6 (3)
C3—C4—C4A—C8A	−19.8 (3)	C4—C4A—C8A—C8	172.3 (2)
C3—C4—C4A—C5	158.0 (2)	C5—C4A—C8A—C1	171.9 (2)
C8A—C4A—C5—C6	1.2 (4)	C4—C4A—C8A—C1	−10.1 (3)
C4—C4A—C5—C6	−176.7 (2)	C7—C8—C8A—C4A	4.8 (4)
C9—O1—C6—C5	12.7 (5)	C7—C8—C8A—C1	−172.9 (2)
C9—O1—C6—C7	−167.9 (3)	N2—C1—C8A—C4A	3.4 (3)
C4A—C5—C6—O1	−176.7 (3)	C11—C1—C8A—C4A	−120.5 (2)
C4A—C5—C6—C7	4.0 (4)	N2—C1—C8A—C8	−178.93 (18)
C10—O2—C7—C8	5.4 (4)	C11—C1—C8A—C8	57.1 (3)
C10—O2—C7—C6	−175.3 (3)	N2—C1—C11—C12	55.1 (3)
O1—C6—C7—O2	−3.5 (4)	C8A—C1—C11—C12	−179.6 (2)
C5—C6—C7—O2	175.9 (2)	N2—C1—C11—Cl1	−68.91 (19)
O1—C6—C7—C8	175.8 (2)	C8A—C1—C11—Cl1	56.4 (2)

### 1-(1-Chloroethyl)-6,7-dimethoxy-1,2,3,4-tetrahydroisoquinolinium chloride Hydrogen-bond geometry (Å, º)

**Table:**

*D*—H···*A*	*D*—H	H···*A*	*D*···*A*	*D*—H···*A*
C1—H1*A*···Cl2^i^	0.98	2.62	3.450 (2)	143
N2—H2*A*···Cl2^ii^	0.95 (2)	2.14 (2)	3.0895 (19)	177 (2)
N2—H2*B*···Cl1	0.92 (2)	2.75 (3)	3.1828 (19)	110 (2)
N2—H2*B*···Cl2	0.92 (2)	2.27 (2)	3.0751 (19)	146 (2)
C11—H11*A*···Cl1^iii^	0.98	2.93	3.767 (2)	144
C12—H12*A*···Cl2^iii^	0.96	2.75	3.710 (3)	174

Symmetry codes: (i) *x*, *y*, *z*−1; (ii) −*x*+2, −*y*+1, −*z*+2; (iii) *x*, −*y*+3/2, *z*−1/2.
